# Volumetric Modulated Arc (Radio) Therapy in Pets Treatment: The “La Cittadina Fondazione” Experience

**DOI:** 10.3390/cancers10020030

**Published:** 2018-01-24

**Authors:** Mario Dolera, Luca Malfassi, Nancy Carrara, Sara Finesso, Silvia Marcarini, Giovanni Mazza, Simone Pavesi, Massimo Sala, Gaetano Urso

**Affiliations:** 1 La Cittadina Fondazione Studi e Ricerche Veterinarie, 26014 Romanengo, Italy; lacittadina@alice.it (M.D.); lucamalfassi@me.com (L.M.); nancy_carrara@hotmail.com (N.C.); sarafinesso@hotmail.com (S.F.); silvia.kant@tiscali.it (S.M.); giobebaliciaylin@gmail.com (G.M.); pavesi.simone@gmail.com (S.P.); salamas@katamail.com (M.S.); 2 Azienda Socio Sanitaria Territoriale della provincia di Lodi, 26841 Casalpusterlengo, Italy

**Keywords:** VMAT, meningiomas, gliomas, trigeminal nerve tumors, brachial plexus tumors, adrenal tumors, rabbit thymomas

## Abstract

Volumetric Modulated Arc Therapy (VMAT) is a modern technique, widely used in human radiotherapy, which allows a high dose to be delivered to tumor volumes and low doses to the surrounding organs at risk (OAR). Veterinary clinics takes advantage of this feature due to the small target volumes and distances between the target and the OAR. Sparing the OAR permits dose escalation, and hypofractionation regimens reduce the number of treatment sessions with a simpler manageability in the veterinary field. Multimodal volumes definition is mandatory for the small volumes involved and a positioning device precisely reproducible with a setup confirmation is needed before each session for avoiding missing the target. Additionally, the elaborate treatment plan must pursue hard constraints and objectives, and its feasibility must be evaluated with a per patient quality control. The aim of this work is to report results with regard to brain meningiomas and gliomas, trigeminal nerve tumors, brachial plexus tumors, adrenal tumors with vascular invasion and rabbit thymomas, in comparison with literature to determine if VMAT is a safe and viable alternative to surgery or chemotherapy alone, or as an adjuvant therapy in pets.

## 1. Introduction

Since the first development of Volumetric Modulated Arc Therapy (VMAT) treatment, VMAT has gained a widespread use in human clinical practice [1]. With this technique, the dose is released to the patients during one or more gantry rotation with simultaneous multileaf collimator aperture optimization and beam intensity modulation. VMAT could obtain better target dose coverage while sparing the organ(s) at risk (OAR) with the possibility of dose escalation trial or a hypofractionated regimen. Moreover, using the VMAT capability of producing inhomogeneous dose distributions, simultaneous integrated boost (SIB) treatments have also been developed. The use of VMAT for various solid tumors and different fractionation schemes has been reviewed [2], showing that VMAT can be considered a safe and effective modality for almost all the investigated pathologies. The results relative to long time toxicity and clinical outcome are sparse as it is a relatively novel technique. Recently, for selected brain metastases, a single fraction high dose treatment (stereotactic radiosurgery (SRS)) has been suggested [3,4] and reviews for both metastases [5] or brain tumors [6] are reported in the human treatment literature. Despite the availability of various papers regarding the hypofractionated radiotherapy in pets for the head and neck tumors [7,8,9,10,11], sarcomas [12] or other sites [13,14,15], few works are devoted to hypofractionated VMAT radiotherapy in pets for the brain meningiomas and gliomas [16,17], trigeminal nerve tumor [18,19], brachial plexus [20], adrenal tumors with vascular invasion [21] and rabbit thymomas [22]. 

Meningiomas represent about 50% of encephalic tumors in dogs and are the most common primary brain tumor in canine medicine [16,22]. Typically, dogs affected are over five years of age and belong to dolichocephalic breeds, whereas boxers show an increased prevalence [16,22]. Surgery is presently considered the treatment of choice for these tumors [8], although radiotherapy and chemotherapy are used for tumors considered unresectable due to their localization or to co–morbidity related risks [23]. Radiation therapy (RT) has also been studied as a definitive or as adjuvant setting after surgery [24,25,26,27,28,29,30].

Gliomas represent a heterogeneous group of neoplasms affecting the central nervous system (CNS) of animals and humans [31]. Radiotherapy with curative or post-surgical adjuvant intent began to play a fundamental role in human medicine glioma treatment, with accompanying improvements in survival time and quality of life [17,32,33,34,35].

Peripheral nerve sheath tumors (PNSTs) are malignant tumors of nerve sheath origin, which develop relatively commonly in dogs [35], apart from tumors affecting the cranial nerves that are relatively uncommon. The trigeminal nerve is most frequently affected [32,36,37,38,39,40]. Limited data is available in the literature regarding trigeminal PNST in dogs [32,37,41]. Brachial plexus tumors are described as heterogeneous neoplasms arising from the cells surrounding the axons of peripheral nerves ranging from spindle cells in fascicles to sheets and cords of pleomorphic cells [35,42]. This kind of tumor spreads both proximally and distally along the nerve and may ultimately involve the spinal cord, causing compression and associated neurological deficits [43]. Metastases are rare, although lung and uveal metastases have been reported [44,45].

Advances in veterinary diagnostic imaging have improved the detection of adrenal masses in dogs [41,46]. Primary adrenal tumors can be functional or non-functional. Among the functional tumors, cortisol-secreting adenomas and adenocarcinomas are the most common types [47], followed by aldosteronomas [48], deoxycorticosterone-secreting tumors [49], catecholamine-secreting tumors [50] and sex hormone-secreting adrenal tumors [51]. Among the non-functional tumors, adenomas and adenocarcinomas are the most frequently diagnosed [47]. Myelolipomas have been occasionally described [52] and adrenal incidentalomas without histological confirmation have also been reported [48,53].

Thymoma is a relatively common disease in rabbits, representing up to 7% of all tumors [54,55,56,57,58,59]. They are generally slow growing with the potential for local invasion and only rarely metastasize [54,59]. Reported therapies include surgery, radiotherapy and adjuvant chemotherapy giving a wide range of survival times [60,61,62,63,64,65,66,67,68]. While traditional radiotherapy techniques have been associated with severe morbidity, in some cases there is a lower acute death rate compared to surgery [69,70]. Most of the previously reported cases used conventional radiotherapy planning techniques, although a few cases were treated with intensity modulated radiotherapy (IMRT) [61,67,69]. 

The “La Cittadina Foundation” results on hypofractionated VMAT therapy for the above described pathology is presented here, showing that this treatment can be considered safe and feasible, and a valuable alternative to standard radiotherapy or to surgery or chemotherapy as an elective, concomitant or adjuvant treatment. 

## 2. Results

In this section were summarized all the results relative to the previously described diseases elsewhere published by our group [17,18,20,22,71]. Following an accurate planning phase, almost all the plans for the various pathologies fulfilled the requested objective for the target and dose constraints for the OAR. Few cases needed a dose reduction to respect the tolerance dose to the OAR. The pretreatment feasibility analysis showed a good agreement between the delivered plan and the calculated one with similar results using both the Mathresolutions Dosimetry Check (DC) and the Scandidos Delta4 (D4) dosimetric system. All the results were above the threshold tolerance level and all the plans were accepted as deliverable. The analysis of the setup reproducibility [17,18,20,22,71] showed that the mean displacements were compatible with the hypothesis of no systematic error with mean displacement along the *X*, *Y* and *Z* axis of the same order of their standard deviation. Amore detailed analysis was reported in the original papers for numeric values, while specific results are reported in the following section.

### 2.1. Meningiomas

Thirty-nine dogs were enrolled [71]. All dogs had a single lesion. Presenting complaints included seizures (21/39), cranial nerve deficits (10/39), altered mentation (7/39) and paresis (8/39). The mean Gross Tumor Volume (GTV) volume at the first computed tomography (CT) simulation time measured was 3.0 ± 1.2 cm^3^ (range 1–8 cm^3^) and the mean Planning Target Volume (PTV) was 4 ± 2 cm^3^ (range 1.5 ÷ 10 cm^3^). During serial clinical examinations, reduction of frequency and/or intensity of seizures (18/21), reduction of detectable cranial nerve deficits (6/10), normalization of mentation as subjectively stated (7/7) and amelioration of the deambulation (8/8) were observed. 

One dog, suffering from an invasive frontal meningioma, experienced a recurrence of presenting complaints 136 days after the end of the irradiation. Magnetic Resonance Imaging (MRI) examination showed a progressive disease with increased volume and mass effect. No other dogs died from the meningioma during the follow-up. Overall one-year and two-year survivals were 84.6% and 74.3%, respectively. A 24-months disease-specific survival rate of 97.4% was estimated. 

Repeated MRI examinations showed variations of irradiated lesions. The categorical assignment to the complete response (CR), partial response (PR), stable disease (SD) and progression disease (PD) groups, resulting from the Combined Response Evaluation System (CRES) post-treatment evaluation during the 24-months follow-up, are reported in Table 1. 

None of the 21 patients affected by seizures at presentation and receiving phenobarbital or topiramate or levetiracetam anticonvulsants interrupted the therapy. Oral administration of methylprednisolone sodium succinate was prescribed to all patients. Corticosteroid dose was increased only for the patient which died of meningioma recurrence, whereas five patients received a stable steroid dose during the follow-up, and the remaining patients received gradually reduced doses.

According to Radiation Therapy Oncology Group (RTOG) toxicity criteria, adverse events potentially related to High Dose Hypofractionated (HDH)-VMAT were limited to grade II in one dog (transient altered mentation, successfully treated with corticosteroids). 

### 2.2. Gliomas

Seventy-two dogs were included in the study [17]. Thirty patients were treated with a palliation protocol, 22 with RT alone and 20 with a combined protocol of RT and temozolomide (TMZ) (RT + TMZ).

The seizure history was collected for all included dogs. The medical records documented seizures, including isolated or multiple episodes, focal or generalized, in 64/72 dogs. The presenting complaints at the first clinical examination were various and included proprioceptive deficits (68/72), altered mentation (49/72), amaurosis (34/72), pacing (23/72), circling (16/72), paresis (7/72) and pain (6/72). During the neurological examination, alterations were detected and graded as absent in 4/72, slight in 19/72, moderate in 25/72 and severe in 24/72 dogs. 

A single brain lesion was detected through MRI in all patients. Four cases were classified as grade I, 10 as grade II, 18 as grade III and 40 as grade IV based on MRI findings. Once surgical biopsy of the brain lesion was performed (four patients), histological results showed oligodendroglioblastoma in one dog and glioblastoma in three dogs. These findings were consistent with a classification of grade IV and confirmed the previous classification carried out based on MRI features. The number of patients for the different grades (I to IV) into the three arms was numerically similar. Mean and standard deviation of tumor volumes were: 5 ± 2 cm^3^ for the CTV volume of the RT group and 5 ± 2 cm^3^ for RT + TMZ group; mean PTV was 7 ± 4 cm^3^ for RT group and 7 ± 4 cm^3^ for RT + TMZ group. Seven dogs were treated with 42 Gy/10 fx, and 15 dogs were treated with 35 Gy/5 fx in the RT arm. Ten dogs were treated with 42 Gy/10 fx, two dogs with 38 Gy/5 fx, four dogs with 35 Gy/5 fx, and four dogs with 33 Gy/5 fx in the RT + TMZ arm. Among the RT and RT + TMZ cases, complete and partial responses were overall observed in 19/24 dogs and 5/24 dogs, respectively, after one year (RT group: partial responses 9/11, complete responses 2/11; RT + TMZ group: partial responses 10/13, complete responses 3/13). Median overall survival time (OST) was lower in the palliation arm (94 days—95% CI 87 ÷ 101) as compared to the RT (383 days—95% CI 276 ÷ 490) (*p* < 0.001) and RT + TMZ arms (420 days—95% CI 280 ÷ 560) (*p* < 0.001), but OST did not differ significantly between the last two groups (*p* = 0.61). Median progression-free survival was 255 days in the RT arm and 345 days in the RT + TMZ arm, with a statistically significant difference between the groups (*p* = 0.027). Following the first year, OST, disease-specific and progression-free survivals in the RT arm were 50.0%, 81.8% and 72.7%, respectively. The subsequent year, OST, disease-specific and progression-free survivals in the RT arm were 40.9%, 72.7% and 72.7%, respectively. In the RT + TMZ arm, at one year, OST, disease-specific and progression-free survivals were 65.0%, 90.0% and 90.0%, respectively. The subsequent year, OST, disease-specific and progression-free survivals in the RT + TMZ arm were 40.0%, 90.0% and 70.0%, respectively. These results are reported in Table 2.

Tumor grade was not correlated with survival (grade I–II, *p* = 0.32; grade II–III, *p* = 0.10; grade III–IV, *p* = 0.68). However, relative tumor volume <5% and clinical presentation without altered mental status were positively correlated with survival (*p* = 0.032). Frontal localization of the tumor was associated with an improved prognosis, but this difference was not significant (*p* = 0.089). Intraventricular localization was inversely correlated with survival (*p* = 0.011). 

All the alive dogs completed the re-imaging follow-up. Twenty-four months later, two subjects were alive and underwent MRI. Magnetic resonance imaging examinations performed during follow-up did not reveal a difference between RT and RT + TMZ. The most common radiological finding for all irradiated patients was a reduction in tumor volume, with progressive shrinkage of the mass and a reduction of the mass effect. A progressive reduction of contrast enhancement in grades I, III (focal) and IV gliomas was observed. However, in one dog, a transient increase in contrast enhancement was observed, despite a reduction in volume. Local recurrences were observed in patients with a previously negative MRI (2/7 dogs), while we observed stable disease in a dog with persistent contrast enhancement. The disease recurrence findings were as follows: increased tumor volume, increased peri-lesional hyperintensity (grade III focal) or lesional hyperintensity (grade III infiltrating), and increased contrast enhancement. 

Another finding observed in four irradiated dogs was diffuse, progressive spinal cord enlargement with diffuse craniospinal thickening, diffuse meningeal contrast enhancement, hydrocephaly and hydrosyringomyelia. All such dogs were euthanized due to progressive worsening of their clinical condition, but none of these dogs underwent necropsy or histologic examination. One dog underwent cerebrospinal fluid (CSF) collection, and cytological examination of the CSF was negative for inflammation but showed neoplastic glial cells. This finding was considered consistent with a possible spreading of the tumor along the neuraxis, but this hypothesis needs further histopathological confirmation. Glioma was the cause of death in all dogs included in the palliation arm. Patient’s recruitment occurred during a four-year period; the mean follow-up was 643 days (range 222–1218 days). Regarding the 22 dogs in the RT arm, 13 deaths were recorded: seven from causes other than gliomas and six caused directly by the tumor itself. Four of these six dogs had the first event reported after RT was local recurrence at 84, 90, 274 and 311 days, followed by death at 99, 103, 306 and 383 days, respectively. 

Among the 20 cases in the RT + TMZ arm, 12 deaths were reported: seven from other causes, while five were directly caused by the glioma itself. Among these five cases, three dogs presented with local recurrence as the first event at 263, 273 and 632 days, followed by death at 312, 318 and 657 days, respectively. A single case showed local recurrence at 620 days but that patient was still alive at 660 days. Two dogs in the RT arm and two dogs in the RT + TMZ arm suffered from the mentioned presumptive spinal spreading at 200 and 214 days (RT), with consequent death at 279 and 274 days, and at 143 and 147 days (RT + TMZ), with consequent death at 180 and 167 days. During the follow-up period, an improvement or normalization of neurological status was observed in all dogs in the RT and RT + TMZ arms and in 7/30 of the patients included in the palliation arm. A reduction in the frequency and/or intensity of seizures was observed in all dogs in the RT and RT + TMZ arms and in 11/30 of the dogs treated under the palliation protocol. None of the patients affected by seizures at presentation and receiving phenobarbital, topiramate or levetiracetam anticonvulsants interrupted the medical therapy. The oral administration of methylprednisolone sodium succinate was prescribed for all patients. Corticosteroid treatment was gradually tapered in the RT and RT + TMZ arms.

Adverse events potentially related to VMAT were limited to Grade II according to the Veterinary Radiation Therapy Oncology Group (VRTOG) toxicity criteria [72], in a single dog presenting with a transient alteration in mentation. The dog was successfully treated with corticosteroids. Minimal radiotoxicity (one Grade II case) and chemotoxicity (two cases of Grade 1 anemia) were also observed.

### 2.3. Trigeminal Nerve Tumors

Seven dogs undergoing VMAT therapy for suspected trigeminal PNST were enrolled [18]. All dogs had a contrast-enhancing trigeminal nerve mass on MRI examination. The imaging characteristics were consistent in all dogs with an extra-axial nodular lesion located at the cerebello-pontine angle involving the intracranial potion of the trigeminal nerve with sharp and regular margins. All masses were strong and uniformly contrast enhancing on contrast-enhanced MRI images. The compression on the brain stem was absent in 4/7, moderate in 2/7 and severe in 1/7. Oedema of the brainstem was detected in two dogs. Slight hydrocephalus was present in one dog. The oval foramen was enlarged in 5/7 dogs. An extracranial extension of the tumor was detected in 4/7 cases; in three of these, the mandibular branch was involved and in another dog all three branches were involved. No biopsies were performed. No relevant events had occurred in the previous medical history and no abnormalities were detected in the serum, CBC or thoracic MRI in any of the investigated dogs. Unilateral renal cysts and splenic nodules suggestive of regenerative hyperplasia were detected in one, and two dogs on abdominal MRI, respectively. CSF examination was normal in all seven cases. The median GTV was 0.4 cm^3^ (range 0.2 ÷ 0.6 cm^3^) and median PTV was 1.1 cm^3^ (range 0.5 ÷ 1.6 cm^3^). All dogs completed the RT course. No acute effects directly referable to the treatment were recorded. All dogs underwent the planned clinical and MRI follow-up examinations. Clinical outcome was characterized by reduction of the central vestibular signs after the first fraction, followed by the disappearance over the two subsequent fractions; on the contrary, the disappearance of facial dysaesthesia and sneezing occurred immediately in all the affected dogs. A reduction of the asymmetry of the masticatory muscles was detected in two dogs after four months, but a persistent asymmetry was subsequently observed in all dogs. Subjectively, a persistently reduced facial, nasal and corneal sensation was observed in all dogs. MRI follow-up examinations revealed CR in one dog, PR in four dogs, and SD in two dogs. Only one dog developed PD, 483 days after the treatment. The signal of the lesion in T2-weighted pulse sequence did not vary significantly in four dogs, whereas in three dogs an increased hyper-intensity in T2-weighted sequence was observed. A homogeneous reduction in contrast enhancement was observed in three dogs, in two dogs a ring enhancement persisted, and in two other dogs, contrast enhancement did not vary significantly. Oedema disappeared in the previously affected dog. No signs directly referable to radiotoxicity were observed. No signs of other cranial nerve or brainstem dysfunction, such as altered behaviour or seizures, were detectable by clinical examination or noticed by the owners. A disease-specific study was not performed because only one dog died of a proven relapse at 523 days while the data for the overall survival analysis were reported in Table 3.

### 2.4. Brachial Plexus Tumors

Ten dogs were enrolled in the study [20]. The presenting complaints at the first clinical examination were: lameness (grade 3/4) and monoparesis (9/10), tetraparesis (1/10), muscular atrophy (7/10), axillary pain (6/10), neck pain (3/10) and Horner syndrome (3/10). All affected dogs had enlarged involved structures of the brachial plexus, hyperintense in T2 weighted pulse sequences and showed a contrast enhancement.

Three dogs had the tumor involved the brachial plexus and proximal nerves, with evidence of a nodular enlargement. Five dogs had the plexus, proximal nerves and roots involved, with nodular enlargement of the plexus and diffuse thickening with hyperintensity in the Short T1 Inversion Recovery (STIR) sequence and contrast enhancement of the affected roots. Two dogs had roots and spinal nerve proximal to the brachial plexus evident, with a nodular enlargement of the affected roots determining a severe intradural compression on the spinal cord. The localization was C6 on the left side in one dog, C6–T1 (3 left, 1 right) in four dogs, C7–T1 in three dogs (2 right, 1 left) and T1–T2 in two dogs (1 right, 1 left). No abnormalities were detected in the serum and CBC.

Mean GTV volume measured at the first CT simulation time was 8 cm^3^ (range 2.7–33 cm^3^) and mean PTV was 114 cm^3^ (range 28–245 cm^3^). The RT prescription was 35 Gy in five fractions given every other day. Only 8/10 plans fulfilled the PTV and the OARs constraints; two plans regarding dogs with nodular enlargement of the root required a recalculation with a reduced prescription (33 and 31 Gy) due to the unacceptable dose delivered to the spinal cord. All dogs completed the RT course. No acute effects directly referable to the treatment were recorded. All dogs underwent the planned clinical and MRI follow-up examinations.During the follow-up period, improvement or normalization of neurological status was observed in all dogs. PR was obtained in all treated dogs. MRI examinations performed during follow-up revealed a reduction in tumor volume and a progressive reduction of contrast enhancement. Slight muscular hyperintensity in STIR pulse sequence within the irradiation field was observed. Local recurrences were observed in 9/10 patients, with a re-presentation of the previous complaints. Mean progression-free survival time was 240 ± 30 days (95% CI 188–291).

Only one dog was alive, 452 days after the end of the treatment, at the time of the writing of the original paper. Mean overall survival was 371 ± 30 days (95% CI, 315–427) and the data for the Kaplan Meier analysis are reported in Table 4 for all the dogs. Euthanasia due to clinical signs related to PNST recurrence was the cause of death for all the other dogs included in this study.

### 2.5. Adrenal Masses

Nine dogs were enrolled [21]. None of the animals exhibited bilateral tumors or metastasis at the time of diagnosis, though in one dog an extra-capsular extension of the tumor with infiltration of retroperitoneal fat was observed. Five dogs had two subsequent MRI examinations including the affected adrenal gland, which highlighted a volumetric progression of the tumor. The mean dorso-ventral diameter of the tumor, at the time of diagnostic CT, was 40 ± 12 mm (range, 23 ÷ 58 mm) and the mean tumor volume was 19 ± 33 cm^3^ (range, 4 ÷ 106 cm^3^). Vascular neoplastic invasion involved the ipsilateral vena phrenicoabdominal and the vena cava abdominals in all dogs. Vena cava obstruction accompanied from severe ascites was observed in one dog. The contralateral gland was normal in 6/9 dogs and reduced in 3/9. Three dogs were affected by CSATs, whereas the other dogs had NSATs. The dogs diagnosed with CSATs started trilostane immediately after the diagnosis, at a dose ranging from 1 to 2.5 mg/kg/24 h. The prescribed mean dose ranged from 30 to 45 Gy in three or five consecutive fractions. The mean GTV volume at the first CT simulation time was 19.4 cm^3^ (range, 4 ÷ 106 cm^3^), and the mean PTV was 27.5 cm^3^ (range, 8 ÷ 113 cm^3^). The dogs were being actively followed; seven deaths were recorded at 337, 489, 890, 912, 1030, 1034 and 1585 days after the end of the treatment, and at the time of original data analysis, the two dogs that were found alive at 1592 and 1632 days after RT were censored. The obtained median survival time was 1030 days. All dogs showed a progressive shrinkage of the tumor during the follow-up and no relapses or metastatic spreads were observed. The overall mean reduction of the dorso-ventral diameter and of the tumor volume were, respectively, 32 ± 7% (range, 23 ÷ 42%) and 31 ± 8% (range, 20 ÷ 45%). Tumor reduction involved both the intravascular and extravascular sides. Survival and reduction data for all the patients were summarized in Table 5. For the dog with ascites resulting from massive vena cava invasion, a reduction in the volume of abdominal fluid was already evident two months after RT, and the ascites was completely resolved at the three-months post-treatment CT/MRI check. In dogs affected by corticol secreting adrenal tumors (CSAT), a progressive reduction of the urine corticoid: creatinine ratio (UCCR) was evident during the follow-up; thus, a progressive reduction in the daily dose of trilostane was possible, with complete withdrawal of the drug in two dogs. One dog developed transient hypoadrenocorticism 15 days after the end of the RT treatment; clinical signs of hypoadrenocorticism were mild, and consisted of depression, polydipsia and diarrhea. No specific treatment was required except for fluid therapy for 5 days. Two dogs displayed anorexia and vomiting during the treatment period, one for two days one and five days for the second; by ultrasound examination, together with cell blood count and chemistry, the diagnosis of mild pancreatitis was posed in terms of acute radiotoxicity. Both the dogs returned to normal with symptomatic therapy. The episodes were not ascribed as ascertained radiotoxicity due to the short duration. Generally, a moderate reduction in contrast graphic opacization in the cranial pole of the kidney ipsilateral to the affected gland, which suggested chronic microvascular damage was observed. No clinical or laboratory findings suggesting acute or chronic renal disease were observed.

### 2.6. Thymoma

Fifteen rabbits were enrolled in the study [22]. Presenting clinical signs included dyspnoea 14/15, exophthalmos 6/15, and anorexia 12/14. One rabbit displaying the thymoma was an incidental radiographic finding and had no noticeable clinical signs. All patients received thoracic radiographs, a CT scan and an MRI scan. All rabbits received a CT guided fine-needle aspiration and were ultimately diagnosed with a Masaoka stage II thymoma. A mediastinal mass was seen in all cases on thoracic radiographs, CT scan and on MRI. Two cases had a small amount of pleural effusion noted. No cases had evidence of pulmonary metastasis. No patients received chemotherapy. Several rabbits were prescribed antibiotics and furosemide during the treatment course. Repeat CT scans with adaptive planning were done on day 5 and 9 of treatment. All rabbits completed their course of radiotherapy without treatment delays or anesthetic complications. Clinical improvement, in those rabbits experiencing clinical signs, was achieved after the first fraction in all rabbits. Reduction of the initial target volumes of greater than 45% and 40% for the GTV and PTV occurred during radiotherapy and are shown in Table 6. The anemia identified on presentation resolved in three of the four cases by three months after completion of treatment. The anemia remained stable during the follow-up period (35.5% hematocrit or HCT) in one case. All three rabbits that were reported to have elevated renal parameters showed a slight decrease in the blood urea nitrogen (BUN) and creatinine values by three months after treatment. Renal function improvement remained stable during the follow-up period in all but one case which at the 12-month follow-up time point showed an increase in renal enzyme parameters, all had fluctuations of measured calcium values during the follow-up period, of the patients that were reported to have increased serum calcium on presentation. During CT examinations taken during the follow-up period, the residual tumor volume was negligible with a complete response being achieved using the response criteria in all cases at their final follow-up CT. Fourteen of the rabbits had complete responses by their 40 day evaluation. One rabbit had a partial response, based on tumor volume at its 40-day and three month follow-up, which then became a complete response on its six month follow up CT scan. No acute or late radiation toxicities were reported. Clinical evaluations at recheck examinations of all rabbits revealed no clinical abnormalities with apparent normal cardio and respiratory function, although specific cardiac evaluation beyond auscultation and pulse evaluation was not done. No abnormalities unrelated to their thymoma were detected in total-body CT scans during follow-up in any of the rabbits. 

Two rabbits died during the follow up period at 677 and 777 days. Three cases were lost to follow up at 729, 729 and 742 days post first day of treatment. The mean follow up time was 801.3 days (range 727 ÷ 90 days) of the 10 rabbits still alive at time of analysis.

## 3. Discussion

The technical difficulties of conformal RT for animals target, with regard to shape irregularity, size, and proximity to critical structures, as well as to markedly heterogeneous structures, were bypassed using VMAT [17,18,20,22,71,72].

The use of a dedicated cradle with a vacuum mattress, a byte block, and the LINAC Cone Beam CT (XVI CBCT) check performed before each session, allowed the authors to deliver a hypofractionated frameless high precision radiotherapy without any invasive device.

Due to the small target volume, the agreement between prescribed and delivered doses and the setup accuracy was considered a key process in the foundation protocol. In this frame, D4 and DC system results analysis demonstrated the treatment feasibility and the good accuracy and setup reproducibility of the immobilization system, as reported in the original papers [17,18,20,22,71]. Radiotoxic effects were rare.

These results suggest that VMAT modality allows higher doses in comparison to standard 3D conformal treatment to be delivered to the target (which might potentially result in a better tumor control probability), with lower doses to the OAR (that could lead to reduced normal tissues complication probability); hypofractionation and Simultaneus Integrated Boost (SIB), where possible, has a better manageability reducing the number of sitting sedation.

Common limitations of the reported studies are the lack of histopathological confirmation and immunohistochemical or molecular tests, small statistical sample and, often, the lack of a necrospy. Below, specific results for the pathologies treated in the clinic are reported.

### 3.1. Meningiomas

The authors’ research [71] showed an overall two-year survival of 74.3%, with an estimated disease-specific survival rate of 97.4%. The local control obtained in this study proved to be better than conformal RT reported results and, more significantly, it is even better than the one obtained with gold standard surgery with resectable patients [73,74]. It is important to note that recent surgical works focusing on improved tumor removal by ultrasonic aspiration and neuroendoscopy reported median survival times of 40–70 months [75,76],which appears to be better than the results of this study. Longer follow-up and higher patient statistics are needed for more accurate comparison, but further stratification is probably necessary for both surgery and the study results to better evaluate small differences.

Using the CRES response assessment, made of volumetric measurements and clinical data assessment, 87.2% of patients showed SD two months after irradiation, 7.7% showed PR and 5.1% CR PR was observed in 48.3% of patients 24 months after irradiation and CR in 17.2%. Among living animals, none of the patients at the 24-month follow-up showed progressive disease.

No animal had been subjected to histological examination of the irradiated lesions, so only hypotheses can be advanced to explain MRI follow up findings. An increase of free water, a reduction of cell density and tumor vascularization could play a role in signal intensity and contrast enhancement variations.

Presumptive diagnosis by imaging is validated by several veterinary medicine specific publications that have assessed 89–100% MRI sensitivity in differentiating neoplastic from non-neoplastic intracranial lesions and 70–96% specificity in identifying tumor type [28,29,77,78,79,80]. Similar results are reported in literature on human treatment [81].

### 3.2. Gliomas

Frameless stereotactic RT in combination with the VMAT technique, solely or combined with TMZ chemotherapy, demonstrated therapeutic efficacy with a consistent improvement in both life quality and expectancy compared to palliation, especially in the absence of any significant adverse effects from irradiation or chemotherapy [17].

The patients in this study underwent serial MRI examinations, so it is reasonable to believe that the recurrence of the tumor would be detected. Additionally, referring veterinarians provided the presumptive causes of death, even if necropsy was not performed. Specific-disease survival was considered reliable for these reasons, even in the absence of histopathology exams, and it was performed considering the deaths related to the tumor as events and the deaths due to other pathologies as censorship. It is noticeable that overall and disease-specific survival times were not statistically different in the RT and RT + TMZ arms. Although one year and two year progression-free survivals were similar in the RT and RT + TMZ groups, median progression-free survival in the RT + TMZ arm (345 days) was significantly longer (*p* = 0.027) [17] than in the RT arm (255 days). This result showed that TMZ can delay disease progression, despite the lack of a change in effective survival time. Temozolomide was chosen for its radiosensitive properties and was also used as an adjuvant to therapy. 

MRI-based criteria were used to categorize tumor type. This enabled the authors to examine the incidence of canine gliomas of various grades; an overrepresentation of grade III and IV tumors was found. 

There was no correlation between survival and MRI grade in both arms of our study. All dogs suffering from grade I gliomas showed a partial or complete response before they died of other causes. These results could raise doubts concerning the effective value in canine medicine of the correlation between the biological malignancy of gliomas and MRI grading criteria, as reported in human medicine [82]. These considerations suggested the importance of further histopathological studies regarding the tumor grade and the response to VMAT treatment.

The most common radiological finding for all irradiated patients was a reduction in tumor volume, with progressive shrinkage of the mass and a reduction of the mass effect. A progressive reduction in contrast enhancement was observed for grade I, grade III focal and grade IV gliomas. These data suggested that the blood–brain barrier exhibited reduced cellularity and regained integrity. Interestingly, the authors observed local recurrences in patients with previously negative MRI findings. Additionally, observed was stable disease in some dogs with persistent contrast enhancement. The hypothesis of poor MRI sensitivity in detection of minimal residual disease could be postulated. Moreover, this is not a pattern of natural glioma spreading in non-irradiated dogs. It is therefore possible that, in irradiated dogs, glioma cells drop from the tumor mass due to the direct effect of irradiation on the stromal burden. However, this hypothesis requires histopathological confirmation.

This paper highlights the importance of considering the relative volume rather than the absolute volume. Dogs with <5% relative tumor volume were associated with more favorable prognoses, in both the RT and RT + TMZ arms. Although absolute tumor size is usually considered in oncology (TNM staging system), relative size seems to have greater prognostic relevance in veterinary practice where more pronounced volume differences are possible. 

### 3.3. Trigeminal

Few studies regarding RT of canine trigeminal PNST are available in the literature. Three dogs were surgically treated and seven underwent no treatment in one of these studies. One dog survived 27 months after surgery, with a range from 5 to 21 months in untreated dogs, and some were euthanized at diagnosis [37]. Another study [44] had four dogs with suspected trigeminal PNST treated by stereotactic radiosurgery (SRS) receiving a median dose of 13.75 Gy (range 12.5 ÷ 17.5Gy) in a single fraction with a median survival of 881 days; follow-up imaging revealed PR in one dog. A retrospective [32] study reported the experience of LINAC cone-based stereotactic radiotherapy (SRT). One or multiple isocenters and arcs were used. Eight dogs were treated with 24  Gy in three fractions. 

This study on VMAT RT, and the study conducted using cone-based SRT, are similar in terms of sample size and inclusion criteria. Moreover, in this prospective trial, a CSF examination ruling out a suspicion of inflammatory disease was performed as an enrolment criterion. During the cone-based SRT study, favorable outcomes were obtained in 5/8 dogs, with 3/8 dogs showing PD. Within this study’s group, 1/7 PD, 2/7 SD, 3/7 PR, and 1/7 CR was obtained. Taking SD, PR and CR together, favorable outcomes in 6/7 dogs, with one dog showing PD were obtained. More importantly, no adverse effects were detected in this group of dogs even if the delivered doses were higher than in the SRT study. Four dogs developed seizures in the study of cone-based SRT. Taken from the data of that study, the reason for seizures was not clear, but a correlation with the RT treatment could be possible. Only two dogs showed brainstem oedema at the initial MRI in this sample. Shortly after the completion of RT, the oedema disappeared. It could be that the oedema was due to a direct inflammation of the brainstem and the early resolution could be an indication of favorable outcome despite the volumetric stability of the tumor.

Concurring with the study of cone-based SRT, a volumetric reduction in most cases was observed. It is difficult to differentiate tumor progression from pseudo-progression caused by tumor necrosis, oedema, and secondary inflammation resulting in an apparently larger region of contrast uptake [82] as reported in human medicine. Only one dog experienced a transient tumor increase, despite an objective and substantial clinical improvement, so it was considered a pseudo-progression in this study. However, this dog showed a progressive reduction of the tumor, but symptoms of suspected tumor regrowth close to the field of irradiation became evident after 12 months. 

Despite the few data reported in literature on cranial nerve PNST treatment, VMAT seems to be an interesting alternative. The sample size considered in this study was too small to recognize the prognostic significance of some MRI findings. Moreover, the natural course of the disease in untreated dogs with suspected trigeminal nerve tumors has not been investigated. 

### 3.4. Brachial Plexus

The radiation dose was selected considering the putative tolerance of the critical structures near the field of irradiation, particularly the spinal cord and in two cases the prescribed dose was reduced in this study [20]. This could represent a bias in the survival curve analysis.

Pain elicited by palpation of the involved region disappeared after RT. The lameness score passed from grade 3/4 in all the patients to 2/4 in three patients and 1/4 for the remaining seven dogs. No corticosteroids were administered. No adverse effects were detected. This could also be related to the small setup margin from the CTV to the PTV that was adopted in this study due to the accuracy and setup reproducibility shown by the displacement analysis for the homemade immobilization system as reported in the original paper [20]. Frameless hypofractionated RT demonstrated therapeutic efficacy with a consistent improvement in both life quality and expectancy compared to literature data [83,84,85,86,87].

It was observed in this study that local recurrence in 90% of treated dogs; mean overall survival was 371 days and progression-free survival was 240 days. These results were comparable with those of the retrospective study [44] concerning tumors with plexus localization but, on the contrary, they are superior in brachial plexus PNST with root localization. Early reports on canine PNSTs described this pathology as a benign and surgically correctable condition [88]. Literature data and the current study disagree with this assumption [83,85,86,87].

Repeated surgery in the retrospective study [44] did not elicit a significant benefit. Among the patients, only one dog was re-irradiated, gaining two more months of good quality of life. The absence of radiotoxic effects in this work, could be related also to the short life span to develop late damage to the spinal cord or a subjective superior tolerability of the spinal cord to radiation. If this assumption was confirmed, a dose escalation trial to assess any correlation between the response and the dose could be performed in future. The most common radiological finding for all irradiated patients was a reduction in tumour volume, with progressive shrinkage of the mass and a reduction of the mass effect.

### 3.5. Adrenal Masses

A volumetric tumor reduction consistent with a partial response (32 ± 7% dorso-ventral diameter, 30.60 ± 7.96% volume) in the treated dogs we achieved [21]. No signs of radiotoxicity nor deaths related to the treatment were registered. 

Endocrine control was reached in the three dogs suffering from CSATs and no ascertain toxicity occurred. Due to the small population, statistical analysis and statistical power was limited. The survival rates were considered just for comparison with published data. The survival analysis was guided by the fact that tumors of the adrenal glands can lead to many systemic effects, also if they are not-secreting. The distinction between disease-specific survival and overall survival was left during data examination in favor of the calculation of the mere overall survival, where the deaths were considered as events for this reason. The resulting median overall survival of 33.8 months was particularly encouraging in comparison with the median survival (17.8 months) reported in literature by a work [89] regarding 20 dogs suffering from NCSATs without adrenalectomy. Moreover, considering the recent and extensive report on 52 dogs having undergone adrenalectomy [90], the technique should be further proven as an alternative to surgery.

Advances in diagnostic veterinary medical imaging have greatly enhanced the capability to identify adrenal masses, including those clinically unapparent detected during imaging examinations for other diseases. The categorization of an incidentally detected adrenal mass (incidentaloma) poses some challenging questions about the potential clinical malignancy and/or production of hormones. No consensus or established guidelines exist for the workup of these veterinary patients due to the lack of clinical trials addressing the course of asymptomatic dogs. The complications that could arise from surgical approach can expose the asymptomatic, incidentally-detected dogs to high risk. Moreover, if endoluminal invasion of the vena cava is observed, the surgical risk increases dramatically, particularly if the venous return to the right atrium is impaired. Peri-operative mortality rates have been reported to range from 19% to 60% [90,91]. However, a direct comparison of the survival times from these various studies is not recommended due to the many differences between the studies and the potential for introducing bias. As reported in literature [92,93], cytology or guided biopsies were not routinely performed due to the high risk and the difficulty to differentiate between benign and malignant lesions also in this study.

Adrenal masses were classified as NCSATs in the study group 6/9. The CT measurements showed a mean value for the maximum tumor diameter greater than 2cm as well as vascular invasion. The presence of growth patterns during subsequent CT examinations and the assessment of vascular endoluminal invasion were considered as criteria for supporting the treatment decision. Three of nine masses were classified as CSATs. UCCR was determined for assessing the need of trilostane therapy and for an early detection of hypocortisolism due to the medical therapy, irradiation or both [94].

Following the RT treatment, normal serum cortisol levels and normal UCCR were achieved allowing the interruption of trilostane administration in two dogs, whereas in the third dog a reduction of the medication was reached. Regarding the endocrine aspects of the CSATs-affected dogs, a good control disease was achieved in all of them. The transient hypoadrenocorticism occurred in one patient could be connected to the high planned dose (42 Gy), so that a dose-dependent endocrine suppression can be supposed [95].

### 3.6. Thymoma

Conventional definitive radiotherapy techniques do not allow adequate sparing of the OAR. Human medicine finds the most common adverse effect is pericardial disease, although precocious or accelerated coronary artery disease is also an important late effect toxicity [65].

The dose fractionation of 40Gy delivered in six fractions in this study was determined by reviewing the literature to estimate the maximum dose that could be delivered to the PTV while staying within the constraints for the OARs. Previously published veterinary study [69], reported radiation-induced pneumonitis (approximately three months after RT) with a minimum, mean, and maximal dose of 0.62 Gy, 6.9 Gy, and 29.4 Gy, respectively. The same data for myocardial heart failure at 12 months has been reported at 3.8 Gy, 25.2 Gy, and 50.4 Gy; the work reported also a median survival time of 313 days in 19 rabbits treated with various radiotherapy techniques and doses; however, three patients died during the first 14 days of RT. The average dose to the heart was of 22 ± 1 Gy in this work [22], lower than the previously reported ones [69]; all the recruited patients were able to complete the protocol without incident and had complete responses by six months and were not found radiation-induced myocardial failure, radiation pneumonitis and alopecia. While these studies cannot be directly compared, this study appears to be more safe and effective than what has been previously reported in the literature. 

Regardless of the surgical approach, surgery for thymoma in rabbits is associated with a reported mortality rate of up to 71% in the perioperative period [63,67]. Suspected paraneoplastic syndromes reported in literature [69,96,97,98] were not found in any of the presenting rabbits in this study, while there were four rabbits that had an elevated calcium level compared to the laboratory’s normal reference range. The authors believe that this was unlikely to represent a true paraneoplastic hypercalcemia as calcium levels in this species vary widely and are dependent on dietary content [98]. Further, during the follow-up period, the calcium levels varied without a discernable pattern making variation due to diet than a paraneoplastic syndrome more likely.

Due to the frequent natural occurrence of thymoma in rabbits this species could be studied to evaluate the radiobiological mechanism of the good response and minor radiotoxicity obtained with this treatment regimen.

## 4. Materials and Methods

Due to the minimal regulation of clinical research in companion animals in the country where the studies were designed and conducted, all the animal protocols used were approved by “La Cittadina Foundation” internal committee on 30/12/2009 (Record number: 07/2009) for the Meningiomas, on 15/03/2010 (Record number: 02/2010) for the Gliomas, on 08/01/2010 (Record number: 01/2010) for the Trigeminal Nerve tumors, on 21/07/2010 (record number: 05/2010) for the Brachial Plexus tumors protocol, on 06/06/2009 (record number 05/2009) for the Adrenal Masses and on 27/12/2011 (record number: 09/2011) for the Thymomas.

“La Cittadina Foundation” has a general protocol for VMAT treatment based on 8 main points:

(1) Use of customized repositioning device
(2) Multimodal volume of interest (VOI) definition
(3) Two-step virtual simulation
(4) Customized treatment plan elaboration
(5) Pre-treatment feasibility analysis
(6) Setup error evaluation
(7) “Intra”-treatment follow up (adaptive radiotherapy)
(8) Specific features for each pathology

### 4.1. Use of Customized Repositioning Device

Due to the small target and organ at risk volumes (OARs), the setup reproducibility plays a fundamental role; in this context, a homemade cradle was realized with an indexing system for the TC and linear accelerator (LINAC) couch. A customized vacuum locked bag is fixed to the cradle for each patient and, if needed in the specific treatment pathology protocol, a bite block is used. 

### 4.2. Multimodal Volume of Interest (VOI) Definition

MRI sequence protocols were established for each specific treatment, and are used to obtain MRI scan also in the treatment setup. A CT scan is performed in the same configuration for treatment planning purposes. CT and MRI scans are coregistered trough an image fusion software. Usually, targets are defined and contoured on MRI scan and then imported on CT scan.

### 4.3. Two Steps Virtual Simulation

The CT scan is performed in the same setup of the LINAC treatment. Three radiopaque markers are embedded into the cradle roughly defining the target isocenter position (setup reference point) in the first virtual simulation step. Second, after the target is contoured, the real target isocenter is identified and a set of displacements from the setup reference point is calculated for the correct LINAC positioning procedure.

### 4.4. Customized Treatment Plan Elaboration

A treatment plan is elaborated with the Monaco (CMS, Elekta) software using a Monte Carlo dose calculation algorithm for each patient. The PTV coverage is considered acceptable for the V_95%_ and the V_107%_ levels (respectively, PTV volume receiving less than 95% and more than 107% of the prescribed dose) of 5% and 7%. Due to the lack of specific pet values, dose constraints are derived by the one defined for humans in TG 101 [99]. Where needed, the simultaneous integrated boost (SIB) treatments are preferred to consecutive boosts.

### 4.5. Pre Treatment Feasibility Analysis

A per patient agreement between the planned and the delivered dose is performed before the beginning of each treatment. Usually, two different systems are used: The Mathresolutions Dosimetry Check (DC) and the Scandidos Delta4 (D4) one. The gamma function [100] with a Dose Agreement (DA) of 3% and a Distance to Agreement (DTA) of 3 mm is used for both the systems for evaluating the results. Moreover, a robust machine quality assurance program has been set as mandatory in the “La Cittadina Foundation” as pet volumes are very small and the required linear accelerator (LINAC) precision is very high. The machine is calibrated using the IAEA TRS 398 [101] protocol and quality assurance checks on the machine is performed to meet standards set forth in TG 142 [102]. A homemade ultra-fast software (Infinity Check, Urso Gaetano, Casalpusterlengo, Italy), freely distributed in Italy, is used to check a huge quantity of LINAC parameter on both static and dynamic treatment. The dose calibration was also periodically confirmed through a dosimetricinter comparison with the Radiation Dosimetry Service (RDS) of the university of Texas MD Anderson Cancer Center (client code 3155) (Houston, Texas, USA) with differences between this center and the RDS always less than 0.5%.

### 4.6. Setup Error Evaluation

Each treatment session began with, a Cone Beam CT (CBCT) acquired through the Elekta XVI system. The obtained scan and the planning CT are coregistered and the shifts are analyzed. The discrepancies between the XVI CBCT and the simulation CT are considered acceptable if the displacement does not exceed 2 mm in any direction. When discrepancies are found to be between 2 mm and 5 mm, table movements are performed in accordance with the XVI CBCT software (Elekta AB, Stockholm, Sweden) results. When discrepancies are found to be more than 5 mm, the patient is repositioned in the cradle and XVI CBCT is repeated to check the patient setup again; if agreement is still not achieved, the whole treatment procedure is repeated starting from the CT simulation. The shift for all the patients of the same pathology are also analyzed to check for the consistency between the setup errors and the setup margins used during the planning phase.

### 4.7. “Intra”-Treatment Follow-Up

Depending on the pathology, a set of clinical examinations and diagnostic CT or MRI are scheduled at fixed treatment sessions. These scans are more frequent for the kind of treatment characterized by a fast response to radiotherapy and, eventually, new treatments with redefined volumes are planned. Other diagnostic examinations are executed if needed. Customized ancillary medicaments are evaluated during the treatment. Radiation toxicities are evaluated clinically and graded according to Veterinary Radiation Therapy Oncology Group (VRTOG) criteria [72,103].

When performed overall, disease-specific and progression-free survival were estimated using the Kaplan–Meier [104] curve analysis method in all the pathologies object of this review. All deaths are considered events while loss to follow-up or being alive at the time of data analysis warrant censoring. Deaths are also grouped as tumor-dependent (recurrence or progression), radiation damage-dependent and radiation damage-independent from the PNSTs. Median overall survival time and the 95% CI are calculated considering the time to the event starting from the end of the radiation therapy.

### 4.8. Specific Features for Each Pathology

#### 4.8.1. Meningiomas

The diagnosis of meningioma was posed based on brain and spine MRI examination, as reported in literature [78,79,80,81,105,106,107]. According to most recent studies, the Magnetic Resonance Imaging (MRI) criteria for presumptive diagnosis of meningioma were: the occurrence of a single solid broad dural based extra-axial mass with distinct margins and the presence of intense and uniform enhancement with a dural tail sign [78,79,80].

The clinical target volume (CTV) encompassed the GTV with supplementary contouring of the dural tail if present. The planning target volume (PTV) was realized by expanding the CTV by 1 mm in all directions. The considered organs at risk (OARs) were eyes, optic pathway, basal ganglia, cerebrum, hypothalamus and hypophysis, brain stem, cerebellum, spinal cord, inner ear, trachea, oesophagus and lungs. The high dose hypofractionated protocol consisted of 33 Gy in 5 fractions delivered over 5 continuous days. All irradiated dogs received 0.1 mg/kg of peri-procedural dexamethasone and anti-inflammatory doses of oral methylprednisolone sodium succinate tapered over three weeks. Phenobarbital or topiramate or levetiracetam were administered to dogs with seizures as presenting complaints.

Specific response evaluation criteria were established to assess the course after irradiation. Categorical attribution criteria were defined as reported in Table 7. One-year and two-year overall and disease-specific survival rates were built according to the Kaplan-Meier method [104].

#### 4.8.2. Gliomas

This study evaluated stereotactic volume modulated arc radiotherapy (VMAT RT) for canine gliomas, alone (RT) and in combination with temozolomide (RT + TMZ), compared to palliation. Overall and disease-specific survival times were estimated.

The presumptive diagnosis of glioma was made based on the brain MRI findings. The MRI criteria were the identification of a solitary intra-axial lesion exerting a mass effect and characterized by an altered T2 signal [106,107].

The clinical-radiological exclusion criteria were:

(a) An acute onset typical of a peri-ictal state, massive cortical involvement of the lesion, localization close to a vascular region, the presence of necrotic and/or hemorrhagic areas: all features that suggested a vascular origin of the lesion [108,109].
(b) A sub-acute onset followed by a progressive worsening, fever and other signs correlated with inflammation, the presence of any predisposing factor for infection in the dog’s history, the recognition of a thin capsule referable to an abscess and concurrent findings of meningitis or ependymitis: these aspects were considered peculiar to infectious and inflammatory alterations [79,110].
(c) Cystic lesions in continuity with ventricles and cisterns, with thin walls and content resembling CSF, that did not display contrast enhancement were arachnoid cysts [111].

Based on MRI findings, and, in particular, the relationship between MRI criteria and tumor grade, the observed gliomas were graded according to the 2007 World Health Organization (WHO) [112] Classification of Tumors of the Central Nervous System and the relationship between magnetic resonance criteria and tumor grade [113].

Neurological alterations were graded according to the modified Glasgow Coma Scale [114].

The schedule of treatment was determined after considering the tumor grade, the volume of the tumor compared to the whole brain volume (relative volume) and the localization. During the second phase of the study, the RT treatment offered was combined with TMZ at 65 mg/m^2^ administered orally six hours prior to each fraction and then for five days monthly for six cycles. The dose of TMZ was derived from the literature with a precautionary adjustment to 65 mg/m^2^ as the daily therapeutic dose is reported to be safe and effective from 60 mg/m^2^ in the dog, up to 200 mg/m^2^ in human patients treated for glioblastoma [115,116,117].

The RT protocol was as follows:

*42 Gy/10 fx:* tumors with a relative volume >10% regardless of grade and localization, diencephalic grade-IV tumors with a volume of 5–10%
*37 Gy/7 fx:* grade IV tumors except diencephalic tumors with a relative volume of 5–10%, diencephalic grade-IV tumors with a relative volume <5%
*33 Gy/5 fx:* tumors classified as grade I, II, or III with a relative volume of 5–10%
*35 Gy/5 fx:* all grade tumors with a relative volume <5% localized near the surface of the cerebrum.

Gross tumor volume (GTV) was defined by contouring the area of signal and structure abnormality in FLAIR or TSE T2-weighted pulse sequences for well-demarcated tumors. The GTV encompassed all areas of signal abnormality in Fluid Attenuated Inversion Recovery (FLAIR) or Turbo Spin Echo (TSE) T2-weighted pulse sequences for infiltrating tumors. Clinical target volume (CTV) was defined by adding the area of peri-tumoral edema to the GTV with the contouring limited by discontinuous neural structures or bony skull. Planned tumor volume (PTV) was defined by expanding the CTV 3 mm in all directions. All irradiated dogs received 0.1 mg/kg dexamethasone peri-procedurally and anti-inflammatory doses of oral methylprednisolone sodium succinate tapered over three weeks. Phenobarbital, topiramate or levetiracetam were administered to dogs with seizures as presenting complaints.

Dogs assigned to the palliation arm received a daily dose of methylprednisolone or dexamethasone; both the doses ranged from 0.5 mg/kg to maximum 2 mg/kg twice daily. Dogs with seizures at presentation in addition to corticosteroid therapy received one or more of the following drugs: Phenobarbital, at variable doses, starting from 2 mg/kg to maximum 4 mg/kg twice daily; topiramate from 5 mg/kg to 10 mg/kg twice daily; levetiracetam 20 mg/kg twice daily or three times a day.

Overall, disease-specific and progression-free survivals were estimated. Associations between survival and tumor grade, tumor localization, relative tumor size and neurologic signs at the time of presentation were tested.

#### 4.8.3. Trigeminal Nerve Tumors

All the dogs that were diagnosed by tests including complete blood cell count (CBC) biochemistry panel and a presumptive, imaging-based, diagnosis of PNST, were included into the study. The MRI criteria to support a presumptive diagnosis of trigeminal PNST were the detection of an enlargement with contrast enhancement of the trigeminal nerve on one side, involving either the intracranial or the extracranial tract across the corresponding neuroforamen, and concurrent wasting of the masticatory muscles of the affected side [118].

MRI examination of the thorax and of the abdomen was performed to exclude concurrent neoplastic diseases. Examination of cerebrospinal fluid (CSF) obtained by a sub-occipital tap was performed to exclude inflammatory intracranial conditions or lymphomatous lesions.

The gross tumor volume (GTV) included all visible tumor and suspect tumor-related contrast enhancement in CT and MRI, and the clinical target volume (CTV) was defined as the GTV with no additional margin. The planning target volume (PTV) [119,120] was obtained with an isotropic expansion of 2 mm margin around the CTV to account for uncertainties of positioning [121]. Relevant OARs were contoured including the brain, brainstem, cerebellum, eyes, inner ears and laryngopharynx [122]. The prescribed dose to the PTV was 37 Gy delivered in five fractions on alternate days. Where possible, the mean dose to one or both inner ears was limited to <16 Gy and the mean dose to the brainstem was limited to <5 Gy.

Neither the severity of neurological signs nor the administration of any symptomatic medical therapy before diagnosis were considered as exclusion criteria. Each dog was video recorded and the results of neurological examinations were registered as a reference for subsequent follow-up examinations. No biopsies were performed.

All the dogs received glucocorticoids at some point during their management. A low dosage of methylprednisolone or prednisolone was administered to the dogs that were not already receiving corticosteroids with a dosage range of 0.2–0.5 mg/kg for the duration of protocol.

The categorical assignment was performed by the radiologist using the following criteria: Patients were ascribed to the complete response (CR) group when the disappearance of all measurable enhancing tumor was observed; Patients were ascribed to the Partial Response (PR) group when a volumetric reduction in the sum of the diameters of the target lesions of at least 30% was found in MRI images, taking the baseline sum as a reference. Patients were ascribed to the Stable Disease (SD) group when a volumetric reduction of less than 30% or an increase of less than 20% was reported in the sum of the diameters of the target lesions; Patients were ascribed to the Progressive Disease (PD) group when either the appearance of one or more new lesions or at least a 20% increase in the sum of diameters of target lesions was observed during MRI scans, taking the smallest sum of diameter in the study as a reference. Overall survival was estimated.

#### 4.8.4. Brachial Plexus Tumors

Inclusion criteria to be admitted to the study were: normal minimum diagnostic tests including complete blood cell count (CBC) and biochemistry panel and a presumptive, imaging-based diagnosis of PNST, assessed by a veterinary expert in imaging diagnostics (MD). No biopsies were performed.

Neither the severity of neurological signs nor the administration of any symptomatic medical therapy before diagnosis were used as an exclusion condition.

Each dog was video recorded, and the results of neurological examinations were registered as a reference for subsequent follow up. Lameness score (0–4) was attributed according to the clinical lameness scoring system for assessment in dogs [123,124]. Neurological alterations were graded according to localization and severity.

The presumptive diagnosis of PNST was developed based on clinical history, neurological and orthopedic examination and spine and plexus MRI [125,126].

The most peculiar clinical aspect of brachial plexus tumor was unilateral forelimb progressive lameness with mono-paresis and muscle atrophy [44]. The MRI criteria supporting PNST diagnosis were either the presence of a nodule in the axilla along the nerves of the brachial plexus or the presence of diffuse or focal thickening of the brachial plexus or its nerve roots [127,128,129].

Brachial plexus tumors were classified as peripheral, of the plexus and proximal nerves and of the roots of the plexus, as described elsewhere [44,129]. GTV was defined by contouring the area of signal and structure abnormality in STIR or Fast Field Echo (FFE) T1-weighted contrast-enhanced pulse sequences, for well-demarcated tumors. For infiltrating tumors, the GTV encompassed all areas of signal abnormality in FLAIR or TSE T2-weighted pulse sequences. Clinical target volume (CTV) was defined by adding 2 cm to the GTV along the involved nerves or plexus, but excluding the spinal cord. Planned tumor volume (PTV) was defined by expanding the CTV 5 mm in all directions. The expansions of the CTV to PTV were limited by the spinal cord but encompassed other nerves proximal to the brachial plexus. The prescribed dose to the PTV was 35 Gy delivered in five fractions on alternate days. The MRI volumetric assessment was identical to the one described in Table 3. Overall, disease-specific and progression-free survival was estimated.

#### 4.8.5. Adrenal Masses

To be admitted to the study the dogs had to meet the following criteria: evidence of one adrenal mass with a dorso-ventral diameter >2 cm with vascular invasion; a normal or reduced (<7.5 mm) contra-lateral adrenal gland as evaluated by computed tomography (CT) and/or magnetic resonance imaging (MRI); a normal pituitary gland evaluated by contrast enhanced MRI. The tumor growth pattern evaluated by serial CT or MRI examinations was considered as adjunctive criteria. Concurrent pathologies and/or administration of medical therapy before diagnosis were not considered as exclusion conditions. The dorsoventral diameter and volumetric measurement of each tumor were performed on CT post-contrast images; vascular invasion was established when multiplanar reconstructed images showed a vascular endoluminal extension of the enhancing mass that was identifiable in all three anatomical planes (i.e., axial, dorsal, and sagittal). Accurate staging of the tumor was carried out using total body contrast-enhanced CT. The diagnosis of hyperadrenocorticism (HAC) was based on the patient history, clinical examination, complete blood count, serum biochemical profile, urinalysis and the results of endocrine function tests: adreno-corticotropic hormone (ACTH) stimulation test, low dose dexamethasone suppression test (LDDST) and urine corticoid: creatinine ratio (UCCR) [130]. Adrenal masses were classified as CSATs when the cortisol concentration was higher than 600 nmol/L or 21.6 g/dL 1 h post-IV administration of ACTH. LDDST was performed in dogs without a positive ACTH stimulation test [131]. Dogs affected by one adrenal mass with a negative ACTH stimulation test, a normal LDDST and normal UCCR (<5), were considered for additional exams to categorize the tumors as functional or not functional. Plasmatic determinations of kalemia and aldosterone were used to exclude the presence of aldosterone-secreting tumors [50,89]. An electrocardiogram (ECG) and non-invasive determination of blood pressure by oscillometry were performed to exclude the presence of pheochromocytomas [90]. Dogs affected by one adrenal mass with a negative ACTH stimulation test, normal LDDST, normokalaemia and aldosteronaemia, normal ECG, and normal blood pressure were categorized as NSAT-affected [89].

Simulation for planned computing and contouring was performed with an abdomen compressor to limit excursion of the viscera during spontaneous breathing. The excursion of the target during normal breathing movements was assessed by four-dimensional (4D) time-resolved, free breathing, retrospective, gated scanning of the affected gland. The gross tumor volume (GTV) was defined as the contour of the tumor including the intravascular extension. The clinical target volume (CTV) was determined by expanding the GTV to the volume of probable tissue invasion, identified as lack of clear boundaries and/or tumor infiltration in the surrounding retroperitoneal fat. Measuring the displacement of the contoured GTV during inspiration and expiration in mms, the margins for the internal motion (IM) was defined. The PTV was contoured as the CTV comprehensive of IM and an additional margin that accounted for the setup uncertainties (setup margin, SM). OAR contouring was planned for each dog, depending on the specific anatomical localization of the target. The effective prescribed dose for each patient varied from 30 to 45 Gy in 3 or 5 consecutive daily fractions depending on the Body Condition Score (BCS) and from the distance between the target and the OARs. Detailed methods with technical specifications are available in the original work [21]. Dorso-ventral diameter and volume measurements were adopted to objectively evaluate the tumor dimensional variation during the follow up. To assess acute renal toxicity, urea nitrogen, creatinine and urinary protein to creatinine ratio were determined before each session and monthly during the 6 monthly follow-ups. To assess acute gastrointestinal toxicity, together with the chemistry, a reporting sheet with a list of gastrointestinal signs or side effects was administered to the owner. Dogs with CSATs had the basal cortisol level determined every 5 days during the first 2 weeks, and the UCCR was measured every 2 weeks for the first 2 months. Subsequently, endocrine tests dictated from the clinical request were performed (ACTH stimulation test, LDDST, UCCR determination). Excessive clinical endocrine control was defined as clinical signs of hypoadrenocorticism and a post-ACTH cortisol concentration lower than 40 nmol/L. Survival was estimated according to the Kaplan-Meier method [105].

#### 4.8.6. Thymoma

Inclusion criteria included rabbits diagnosed with thymoma based on either cytology or histology that received no prior medical or surgical intervention except supportive care. All animals were staged according to the Masaoka staging system used in humans, as no veterinary staging system was available at the time of the original paper [132]. Staging tests included complete blood counts, serum biochemistry panels, thoracic radiographs, full body computed tomography (CT) and full body magnetic resonance imaging (MRI) [133].

The clinical target volume (CTV) was defined as all gross disease as visualized on the planning CT. The planning target volume (PTV) was created by expanding the CTV by 3 mm in all directions. The PTV was further divided into a full dose PTV and a partial dose PTV for the portion of the PTV that was directly in contact with the heart, allowing a forced maximum dose of 36Gy to that region. A total dose of 40Gy delivered in 6Gy fractions over a 11-day period was planned with rabbits to be treated on days 1,3,5,7,9 and 11.Tumor volumetric response was scored using the scheme in Table 3 [22,134,135]. Survival analysis was done using the Kaplan-Meier method. Since no necropsy exams were done, all animals that died were considered dead of disease.

## 5. Conclusions

The current work shows that VMAT hypofractionated RT is a feasible and effective therapeutic option for different pet pathologies. The treatments were generally well tolerated; the rare or absent radiotherapy induced toxicities seem to suggest dose escalation trials while the good clinical results recommend the VMAT as a viable and safe alternative to other standard approaches. Often, these studies suffer from a limited number of patients and a greater statistical sample would confirm the results better.

The authors believe VMAT could be used successfully in almost all the small pet radiotherapy treatments, and are under analysis. Additionally, other pathologies that were prevented in the past, by high toxicities due to the OAR tolerance doses and target proximity are being investigated. 

## Figures and Tables

**Table 1:** Outcome according to the Combined Response Evaluation System (CRES): the categorical attribution of the patients during the 24-months follow-up.

	2 Months	4 Months	6 Months	12 Months	18 Months	24 Months
Complete Response	2	2	2	3	3	5
Partial Response	3	12	17	18	19	14
Stable Disease	34	23	16	12	10	10
Progressive Disease	0	0	1	0	0	0
Number of alive patients	39	37	36	33	32	29

**Table 2:** Survival in the RT and RT + TMZ arms.

	One Year			Two Years		
	Overall Survival	Disease-Specific Survival	Progression-Free Survival	Overall Survival	Disease-Specific Survival	Progression-Free Survival
RT	50%	81.8%	72.7%	40.9%	72.7%	72.7%
RT + TMZ	65%	90%	90%	40%	90%	70%

RT, radiation therapy; TMZ, temozolomide.

**Table 3:** Data regarding the Kaplan Meier analysis with indicated the cause of death or the type of censoring and the type of response.

Dog	Censored/Cause of Death	Survival at the End of the Study (days)	Response
#1	Censor	1187	SD
#2	Censor	754	PR
#3	Euthanasia	523	PR-PD
#4	Censor	389	CR
#5	Gastric dilatation volvulus	952	SD
#6	Hemorrhagic gastroenteritis	649	PR
#7	Car Accident	313	PR

**Table 4:** Data for the Kaplan Meier analysis. The only censored data is for the 10th patient.

Dog	Overall Survival (days)	Progression Free Survival (days)
1	179	98
2	265	141
3	345	201
4	377	227
5	381	233
6	397	239
7	418	282
8	423	288
9	446	295
10	482	392

**Table 5:** Classification of the selected dogs with volume reduction and survical data.

Dog	Classification	Volume Reduction (%)	Survival (days)	Censored/Cause of Death
1	NSAT	20	337	Pulmonary tromboembolism
2	CSAT	35.48	1030	Intracranial neoplasia
3	NSAT	35.58	890	Cerebral stroke
4	NSAT	24.53	1034	Analsac adenocarcinoma
5	CSAT	34.85	1585	Cardiac and renal failure
6	NSAT	24	912	Cardiac failure
7	NSAT	45.19	1632	Censored (alive)
8	CSAT	30.95	1592	Censored (alive)
9	NSAT	24.78	489	Vehicular accident

**Table 6:** Target volume reductions during treatment as determined by CT contoured volumes.

	Pre-Treatment Volume (cc)	Treatment Day 5 Volume (cc)	Treatment Day 9 Volume (cc)
GTV	19 ± 4	9 ± 3	6 ± 2
PTV	24 ± 7	13 ± 6	10 ± 4

GTV, Gross Tumor Volume; PTV, Planning Target Volume; CT, computed tomography.

**Table 7:** Combined Response Evaluation System (CRES): MRI volumetric assessment implemented with clinical evaluation and the need of corticosteroid administration.

	MRI Volumetric Assessment	Corticosteroids Requirement	Clinical Status
**Complete Response** **(CR)**	disappearance of all enhancing tumor	patient not receiving steroids	stable or improved clinical status
**Partial Response** **(PR)**	≥30% decrease in tumor volume	stable or decreased steroids dose	stable or improved clinical status
**Stable Disease** **(SD)**	≤30% decrease or ≤20% increase in volume	stable or decreased steroids dose	stable or improved clinical status
**Progressive Disease** **(PD)**	≥20% increase in tumor volume	stable or increased steroids dose	clinical deterioration

MRI, Magnetic Resonance Imaging.
